# O.6.1-10 Associations between physical education anxiety and physical fitness through five years

**DOI:** 10.1093/eurpub/ckad133.269

**Published:** 2023-09-11

**Authors:** Timo Jaakkola, Sarah Costigan, Arto Gråsten, Mikko Huhtiniemi

**Affiliations:** University of Jyväskylä, Finland; University of Jyväskylä, Finland; United Arab Emirates University College of Education (CEDU) Physical Education Department; mikko.huhtiniemi@jyu.fi; University of Jyväskylä, Finland

## Abstract

**Purpose:**

Physical education is an important context to contribute students’ physical activity in childhood and adolescence. However, negative physical education experiences may have a long-lasting association on students’ willingness to participate in physical activity. The associations between students’ anxiety in physical education and physical fitness have not been previously investigated. The aim of this longitudinal study was to analyse associations between physical education anxiety and physical fitness through five years.

**Methods:**

Participants of this study included 1147 Grade 5 students (565 males) (mean age: 11.27(0.32) years) at the baseline measure. These participants were measured annually between Grades 5 to 9. The Finnish version of the Physical Education State Anxiety Scale (Barkoukis, 2007) was used to measure cognitive anxiety in physical education. Physical fitness was measured by 20meters shuttle run test (cardiorespiratory fitness) and curl-up and push-up tests (muscular fitness). Sex, BMI and peak height velocity was used as covariates in the analysis. Associations between study variables were measured by the Random Intercept Cross-Lagged Panel Model using Mplus statistical software.

**Results:**

Between level results (squared multiple correlations) of the analysis demonstrated that cognitive anxiety was related to cardiorespiratory (-.32) and muscular fitness (-.34) over five years. Additionally, sex (-.12; girls higher anxiety), BMI (.16) and peak high velocity (-.23) had significant associations with cognitive anxiety.

**Conclusions:**

This study revealed that cognitive anxiety in physical education has an influence on student’s physical fitness longitudinally. These findings were similar between anxiety and cardiorespiratory fitness and between anxiety and muscular fitness. Results demonstrate that negative physical education experiences have an association on cardiorespiratory and muscular fitness from late childhood to adolescence. Constructs of the Self-determination theory (basic psychological needs) and Achievement goals theory (task orientation) are helpful when implementing positive pedagogy in physical education.

**Support/Funding Source:**

Finnish Ministry of Education and culture funded this study.

